# Beyond transmission: Dire need for integration of nutrition interventions in COVID-19 pandemic-response strategies in Developing Countries like Pakistan

**DOI:** 10.12669/pjms.36.COVID19-S4.2784

**Published:** 2020-05

**Authors:** Rubina Hakeem, Muhammad Adil Sheikh

**Affiliations:** 1 Prof. Dr. Rubina Hakeem, Ph.D. (UK) RD (UK). Fellow of Association for Nutrition (FAfN), UK Principal, RLAK Govt. College of Home Economics, Karachi, Pakistan; 2 Muhammad Adil Sheikh, Clinical Assistant Professor Division of Hospital Medicine Department of Internal Medicine University of Michigan Ann Arbor, Michigan. US

**Keywords:** Infections, COVID19, Nutrition, Immunity

## Abstract

Synergistic associations between infection and nutrition are well known. Impact of nutrition interventions on the outcomes have been scientifically assessed and reported. The role of nutrition in limiting the infection related morbidity and mortality does not appear to be a debatable question but nutrition interventions do not appear to be an essential part of current COVID-19 management strategies. Given the nature of pandemic and lack of organism-specific evidence, variability in nutrition interventions and lack of nutrition interventions is not unexpected. However, delay in realization of the crucial need of nutrition interventions to limit the immediate and long term outcomes at personal and community level may aggravate health related issues that can have long term impact on quality of life and economy. Due to existing undernutrition and lack of nutrition related awareness and competence, need for timely and appropriate interventions is much more critical for developing countries. This manuscript highlights the need and feasibility of various nutrition interventions to assure optimum quality of life during and after COVID-19 pandemic. Available evidence provides enough guidance for nutrition interventions that are safe and promise to accrue various degrees of benefits with almost no likelihood of harm. Nutrition interventions suggested by author are: 1) population level efforts for promoting better use of existing resources; 2) quicker augmentation of nutrition status of high risk people and non-hospitalized cases by use of supplement and individualized guidance and 3) nutritional support of sever case by timely and adequate enteral and parenteral feeding.

## INTRODUCTION

COVID-19 is a highly infectious disease caused by infection with a new strain of coronavirus identified in late 2019. Absence of vaccination and evidence-based treatment regimes, rapid escalation in cases; and need for hospitalization and mechanical ventilation of severe cases has made its management extraordinarily challenging.^1^,^2^

The main strategy suggested by experts to deal with the pandemic to flatten the curve by slowing down the rate of transmission, specially to people at high risk of severe impact. Personal hygiene, voluntary social distancing and mandatory restrictions to distance people are the measures being stressed to reduce the rate of transmission.^3^ Still a vast majority of world’s population is at risk of getting the infection at some stage of the pandemic.^4^ Once infected the host’s ability to limit the activity of the virus and possession of sufficient physiologic resources to preserve vital functions during the period of fight between virus and the body are the key factors that makes a difference on the impact of the infection.^5^-^7^ Enhancing an individual’s ability to fight off infection without exhausting physiologic resources could be an additional strategy that can provide support in reducing the burden of COVID-19 severity.

This manuscript highlights the available evidence to identify nutrition interventions that have potential to alleviate the negative impact of COVID-19 pandemic on developing countries like Pakistan.

## METHODS

To explore the potential of nutrition interventions in supporting the management of COVID-19 available evidence regarding mechanism of COVID-19 infection and its clinical course sequel was retrieved form “PubMed”. All the evidence, and guidelines by professional association and expert’s opinions about role of nutrition in managing COVID-19 was also studied. Evidence about role of nutrition in managing similar viral infections and similar pathophysiology was also reviewed. Search terms used included: “COVID-19 AND Nutrition”, “COVID-19 AND food” “COVID-19 AND Pakistan”; “Viral Infections AND Nutrition” “Viral Infections AND food”.

For estimating the feasibility and effectiveness of possible nutrition intervention in Pakistan information was retrieved from research databases and publications of international agencies and government of Pakistan. Search terms used included: “Viral Infections AND Pakistan” “NUTRTIONAL STATUS AND Pakistan” “Diet quality and Pakistan” “Nutrition AND Pakistan”.

## RESULTS

### The need for nutrition interventions

Evidence overwhelmingly supports the notion that nutrition can influence any person’s ability to fight off viral infection.^5^-^7^ Status of food insecurity in Pakistan^8^ reflects on the inability of a large proportion of population to consume adequate diet. Food habits and perceptions of good food points toward high risk of consumption of unhealthy diets even among food secure groups. People with obesity, hyperglycemia, and dyslipidemia are observed to have more severe consequences of COVID-19 infection and if such persons have protein and micro nutrient deficiencies their ability to survive the infection is further reduced.^9^-^11^ Co-existence of high rates of malnutrition and chronic disease presents a grave situation in terms of Pakistani population’s nutritional fitness to combat viral infection and points towards possibilities of significant dividends from appropriate and timely nutrition interventions.

### Population level nutrition interventions

Development of vaccine to prevent COVID-19 could take more than year and even when developed its availability to all is not guaranteed. Recent reports of re-occurrence of infection in infected patients diminish hope for assured natural immunity. The high rates of morbidity and mortality highlight the need for additional public level strategies to support the immune system, so that the impact of the infection can be reduced.

**Fig.1:**
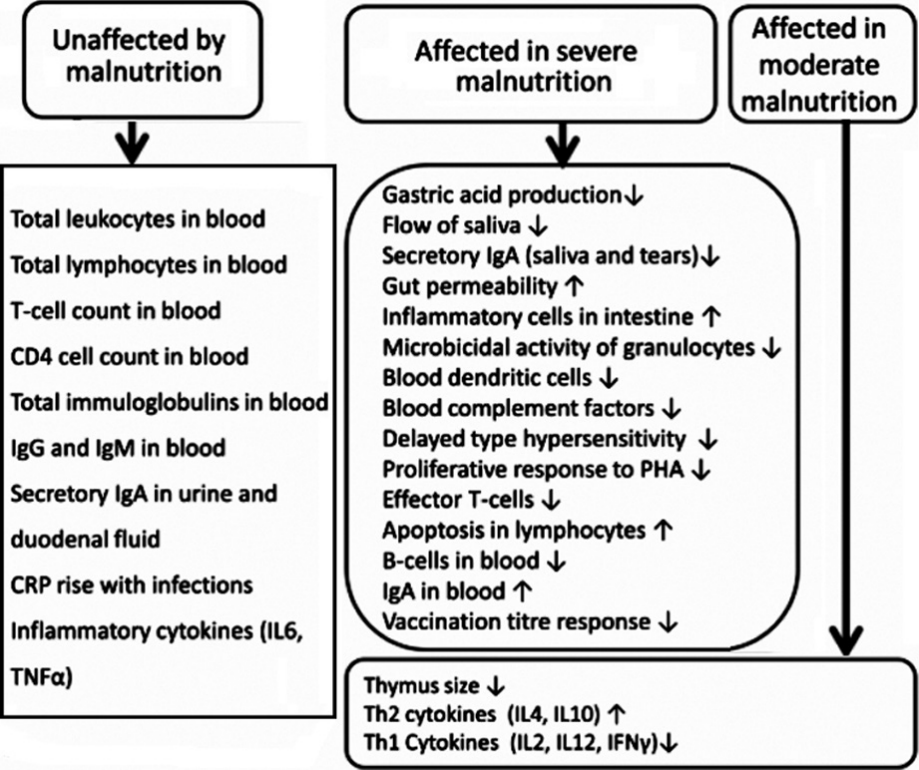
Summary of immune parameters affected and not affected by malnutrition.^12^

Impact of nutrition on immunity has been acknowledged for decades and mechanism of actions have been presented by many researchers. One simple presentation is quoted in Fig.1^12^

Moderate-quality evidence exists from both human intervention and observational studies to suggest that diet and individual nutrients can influence systemic markers of immune function and inflammation;^13^ Micronutrient deficiencies or suboptimal levels negatively affect immune function and lead to decreased resistance to infections.^6^

Lack of national and population level data about food and nutrient intake limits the opportunities for accurate assessment of micronutrient status of Pakistani population. Evidence available from small studies and food balance sheets leaves no doubts about presence of multiple micronutrient deficiencies in a large section of the population.^14^,^15^ Limitations of public guidance on diet and low level of nutrition competence diminishes hopes of proportionate improvement in micronutrient status with income levels. Thus micronutrient deficiencies are likely to be widespread and need for correcting these deficiencies to enhance the immune status of people is great. Hydration and nutrition are stated as important weapons in fight against COVID-19.^1^,^16^-^18^

In Pakistan, Micronutrient status of a high proportion of people can be improved simply by modifying food choices, food preparation methods, meal planning and within household food distribution. A brief list of required modifications is given in Table-I for many of these modifications people only needs to be made aware while for others motivation is needed to change cultural norms. The alarming and long term threats posed by the pandemic may provide enough motivation to change the food preparation and food distribution practices. Any degree of change in the positive direction would give at least some improvement in immunity and health and long term benefits for public health and national economy. For creating awareness short, technically sound and culturally befitting messages needs to be developed and robustly disseminated to masses in local languages as part of “fight against COVID-19” campaigns. For example, instead of just saying staying home, message could be extended to say *“Stay home, wash hands and strengthen your immunity by healthy diet, exercise and relaxation”*; “*no matter how healthy you are, for stopping entry of the virus you need to stay at home and wash hands; but once the virus enters your body, the strength of body’s defense system decides the outcome and by eating healthy we can strengthen our immune system”*. Descriptions of healthy diet can be given in more detailed messages.

**Table-I:** Modification needed in food related habits of Pakistanis to make their diets healthier.

Objective	Examples
↑ Vit C	Guavas, Amla,
↑ Vitamin D	Egg, beef, beef liver, fatty fish
↑ Complete Proteins	Meat, egg, rice/Wheat eaten with lentils
↑ Zinc	Beef, whole wheat, beans, nuts
↑ Omega 3 fatty acids	canola oil, low erucic acid mustard oil, soya bean oil
↑ Fresh food	Fruit, salads, minimally cooked vegetables
↓ Overcooked food	Vegetable bhujias
↓ Sugary food	Pakistani sweets (Mithais), candies, desserts
↓ Ultra processed food	Crisps, biscuits, refined floor
↓ biased food distribution	Instead of giving Meat and fresh food given to males distribute evenly

Mobile-health-interventions are observed to be effective in promoting behaviours change in many health and nutrition related behaviors in lower income groups in other countries.^19^,^20^ In Pakistan also text messages are observed to bring behavior change in uptake of health care.^21^,^22^

For optimizing immune status, Calder et al on the basis of recent review recommend a multivitamin and mineral supplement that supplies the basic micronutrient requirements (e.g. RDA) for vitamins and minerals in addition to the consumption of a well-balanced diet. They allow supplementation above the RDA, but within recommended upper safety limits, for specific nutrients such as vitamins C and D and intake of 250 mg EPA + DHA per day.^23^ Suggesting the use of supplements may not be suitable for whole of Pakistani population but should be considered for higher risk people. Nevertheless, such recommendations can guide identifications of local foods that are rich resources of these nutrients.

In addition to consumption of a healthy diet, provision of nutrition support by concentrated food sources and supplements to confirmed or suspected cases of COVID-19 can help in reducing hospital visits and hospitalizations. Use of hydration solutions, concentrated forms of high-energy, high-protein, micro-nutrient rich diet and use of nutrition supplements may be needed for symptomatic non-hospitalized patients.^1^ In such situation, ideally they should have access to nutrition care by qualified dietitians so that they can get individualized dietary advice. In low resource populations like Pakistan, aggressive nutrition interventions among these people is an important gateway of opportunity to reduce the severity of disease, number of hospitalizations and burden on health care resources.

### Nutrition Interventions for hospitalized

Regarding nutrition care of COVID-19 in health care facilities ASPEN states: *“Nutrition intervention and therapy needs to be considered as an integral part of the approach to patients victim of SARS-CoV-2 infection in the ICU setting, internal medicine ward setting as well as in general healthcare.”* because *“Optimal outcome can be improved implementing adherence to recommendations to ensure survival of this life-threatening disease as well as better and shorter recovery”*. ASPEN recommends nutrition screening of all high-risk patients of CVOID-19 and patients found to be malnourished should seek diet counselling form expert professionals, sufficient supplementation with vitamins and minerals, oral nutritional supplements (ONS), early enteral feeding and parenteral nutrition where provision of adequate amount of required feeds is not feasible via enteral nutrition.^24^ Most of these recommendation can be met in Pakistan hospitals if efforts are made to employ qualified dietitians and assure steady provision of formulas and feeds. Development of guidance on using safe alternatives for commercial formulas and toolkits to guide nutrition screening assessment and calculation of feeds can facilitate optimum nutrition care to hospitalized patients.

## DISCUSSSION

This paper has reviewed available evidence that could assist in estimating the potential of nutrition in reducing the impact of COVID-19 infection. The findings strongly support the role of nutrition in reducing COVID-19 related morbidity and mortality in any population but opportunities for utilization the potential of nutrition are much greater in malnourished people.

As low resource countries have very limited ability to manage critically ill cases of COVID-19, there is a dire need for improving the immunity of people by all available approaches including nutrition. Integration of nutrition interventions in COVID-19 pandemic-response strategies in urgently required in developing countries like Pakistan.

Nutrition interventions suggested by author are: 1) population level efforts for promoting better use of existing resources; 2) prompt improvement of nutritional status of high risk people and non-hospitalized patients by use of supplements and individualized guidance and 3) nutritional support of severely ill patients through timely and adequate enteral and parenteral feeding.

Similar concerns and suggestion have been shared by other nutrition experts from relatively more developed countries.^18^,^23^,^25^,^26^
